# Arterial Spin Labeling Imaging Assessment of Cerebrovascular Reactivity in Hypertensive Small Vessel Disease

**DOI:** 10.3389/fneur.2021.640069

**Published:** 2021-06-30

**Authors:** Bo-Ching Lee, Hsin-Hsi Tsai, Abel Po-Hao Huang, Yen-Ling Lo, Li-Kai Tsai, Ya-Fang Chen, Wen-Chau Wu

**Affiliations:** ^1^Department of Medical Imaging, National Taiwan University Hospital, Taipei, Taiwan; ^2^Graduate Institute of Clinical Medicine, College of Medicine, National Taiwan University, Taipei, Taiwan; ^3^Department of Neurology, National Taiwan University Hospital Bei-Hu Branch, Taipei, Taiwan; ^4^Department of Neurology, National Taiwan University Hospital, Taipei, Taiwan; ^5^Division of Neurosurgery, Department of Surgery, National Taiwan University Hospital, Taipei, Taiwan; ^6^Institute of Medical Device and Imaging, National Taiwan University College of Medicine, Taipei, Taiwan

**Keywords:** arterial spin labeling imaging, intracerebral hemorrhage, hypertension, cerebral small vessel disease, cerebrovascular reactivity

## Abstract

**Objective:** Cerebrovascular reactivity (CVR) represents the phenomenon where cerebral vessels dilate or constrict in response to vasoactive stimuli. CVR impairment may contribute to brain injury due to cerebral small vessel disease (SVD). We aimed to determine the CVR in hypertensive intracerebral hemorrhage (ICH) and to identify its vascular dysfunction.

**Methods:** A total of 21 patients with spontaneous hypertensive ICH (strictly deep or mixed deep and lobar hemorrhages, mean age 62.5 ± 11.3 years) and 10 control subjects (mean age 66.1 ± 6.0 years) were enrolled for CVR measurement at least 3 months after the symptomatic ICH event. Each participant underwent a brain MRI study, and CVR was calculated as the cerebral blood flow (CBF) reduction using arterial spin labeling (ASL) between baseline and 10 min after an intravenous dipyridamole injection (0.57 mg/kg). Traditional MRI markers for SVD were also evaluated, including cerebral microbleed, white matter hyperintensity, lacune, and MRI-visible enlarged perivascular space, which were used to determine the total small vessel disease score.

**Results:** Compared to control subjects, hypertensive ICH patients showed reduced CVR in the basal ganglia (CBF reduction 22.4 ± 22.7% vs. 41.7 ± 18.3, *p* = 0.026), the frontal lobe (15.1 ± 11.9 vs. 26.6 ± 9.9, *p* = 0.013), and the temporal lobe (14.7 ± 11.1 vs. 26.2 ± 10.0, *p* = 0.010). These differences remained significant in multivariable models after adjusting for age and sex. Within ICH groups, the CBF reduction in the basal ganglia was significantly correlated with the total small vessel disease score (R = 0.58, *p* = 0.006), but not with individual MRI markers.

**Conclusion:** Patients with advanced hypertensive SVD demonstrated impaired vasoconstriction after dipyridamole challenge in the basal ganglia and the frontal and temporal lobes. Our findings provide safe approaches for whole-brain CVR mapping in SVD and identify a potential physiological basis for vascular dysfunction in hypertensive SVD.

## Introduction

Hypertensive small vessel disease (SVD) is the most common etiology of non-traumatic spontaneous intracerebral hemorrhage (ICH) and has significant morbidity and mortality around the globe (1, 2). The underlying small vessel pathology in hypertensive SVD is characterized by fibrinoid necrosis and lipohyalinosis of small penetrating arteries that directly stem from large vessels in deep cerebral regions, such as the basal ganglia and thalamus (3). In addition to symptomatic ICH, hypertensive SVD can result in the occurrence of cerebral microbleeds (CMBs) and other non-hemorrhagic presentations, including deep lacunar infarcts and white matter hyperintensities (WMH), which typically can be detected in conventional MRI (4).

Recently, a few studies using neuroimaging techniques have demonstrated that hypertensive SVD could be involved in not only deep but also lobar areas, causing a clinical presentation of mixed lobar and deep hemorrhages in advanced stages (5, 6). However, most of the studies on hypertensive SVD are still focusing on analyzing conventional MRI markers, including the distribution and number of CMBs, lacune, WMH, and MRI-visible enlarged perivascular space. These markers could only reflect the end-stage parenchymal change from damaged small vessels.

As a complementary technique to steady-state neuroimaging markers, functional neuroimaging to assess cerebrovascular reactivity (CVR) can represent the phenomenon where cerebral vessels dilate or constrict in response to vasoactive stimuli. CVR measure could complement steady-state perfusion parameters, such as cerebral blood flow (CBF) and cerebral blood volume, by providing mechanistic insights into understanding vascular autoregulation. In hypertensive SVD, regional reduced CVR may lead to unmet cerebral perfusion demand, which ultimately predisposes these patients to damaging ischemia, especially in deep brain regions (7, 8).

To date, there is poor understanding of the changes of CVR in patients with remote spontaneous ICH, which is a group of patients that mostly harbors severe hypertensive SVD. Thus, it would be of interest to explore the pathophysiology of SVD-related injury. We hypothesize that patients with hypertensive SVD would have impaired CVR in both deep and cortical vessels.

In this study, we applied intravenous dipyridamole injection as a vaso-constrictive stimulus and used arterial spin labeled (ASL) perfusion imaging to measure the cerebral perfusion reduction before and after dipyridamole injection. Our primary aim is to compare the severity and region of reduced CVR between hypertensive ICH patients and healthy controls. As a secondary objective, we aimed to assess the association between regional CVR and conventional MRI SVD markers.

## Materials and Methods

### Patient Selection and Clinical Data Collection

We prospectively recruited patients who had symptomatic spontaneous hypertensive ICH (*n* = 21) from National Taiwan University Hospital (NTUH). All patients with hypertensive ICH were in the chronic stage (>3 months). We excluded patients with potential causes of secondary hemorrhage, including trauma, structural lesion, brain tumor, severe coagulopathy due to systemic disease or medication, and those who had ischemic stroke with hemorrhagic transformation. Patients were also excluded if they had a history of cardiac disease (coronary heart disease, congestive heart failure, or cardiac arrhythmia) or asthma.

Hypertensive SVD was determined based on the topography of hemorrhagic lesions (both hematoma and microbleeds) using susceptibility-weighted imaging (SWI). We included strictly deep ICH patients (deep ICH with or without deep CMBs) and patients with mixed ICH (a combination of mixed lobar and deep ICH and CMBs) (5, 9). Ten healthy controls without known neurological or cardiovascular diseases were recruited from the same institution. Clinical demographic information was recorded by a trained study nurse (author Y.-L. Lo) through interviews with each participant and reviews of their medical records. The following parameters were collected systemically: age, sex, body mass index, presence/absence of hypertension, classes of antihypertensive medication, diabetes mellitus, hypercholesterolemia, history of ICH and ischemic stroke, and values of creatinine clearance. Cognitive function was evaluated in each participant by the Mini-Mental State Examination and the Montreal Cognitive Assessment (10).

### Standard Protocol Approvals, Registrations, and Patient Consents

This study was performed with the approval of the institutional review board (201811003RINB) at NTUH and in accordance with their guidelines. Written informed consent was obtained from all participants or their family members.

### Image Acquisition

Brain MRI was performed on all participants using a 1.5-T scanner (MAGNETOM Aera, Siemens, Erlangen, Germany), including T2-weighted turbo spin echo, fluid-attenuated inversion recovery (FLAIR), susceptibility-weighted imaging (SWI), diffusion-weighted imaging, time of flight angiography, and three-dimensional T1-weighted gradient echo sequences. For the assessment of CBF reduction, 0.57 mg/kg of dipyridamole was intravenously injected for 4 min as a vaso-constrictive stimulus. Regional CBF was measured before and 10 min after dipyridamole injection using pulsed ASL (bolus duration = 0.5 s, inversion time = 2.4 s) and three-dimensional gradient-and-spin-echo readout (TR = 4 s, TE = 16.2 ms, in-plane matrix = 64 × 64, 40 slices, voxel size = 3.1 × 3.1 × 3 mm^3^, scan time about 4 min).

### Image Analysis

Image data were processed using PMOD Version 4.1 (PMOD Technologies Ltd., Zurich, Switzerland). ASL images were converted to CBF maps using published procedures (11). The CBF map and 3D T1-weighted images were converted to NIfTI file format. After rigid co-registration of the matched CBF map and 3D T1-weighted images, PMOD software spatially normalized the 3D T1-weighted images into standard Montreal Neurological Institute space based on the anatomical automatic labeling (AAL) atlas (12). The maximum probability atlas (AAL-VOI) was then generated and segmented the ASL images into major brain regions. The processed ASL images and atlas-based volume of interest (VOI) were manually examined for accurate co-registration and segmentation. Regional analyses were then performed in the targeted volumes, including the frontal, temporal, parietal, and occipital lobes, basal ganglia, cerebral white matter, and pons. Baseline relative CBF in regions of interest was corrected using cerebellum CBF (bilateral cerebellum for healthy control and ipsilateral cerebellum for ICH group to avoid crossed cerebellar diaschisis). As for CVR measurement, the percentage of change in the CBF temporal signal before and after dipyridamole injection was calculated and analyzed without cerebellum CBF correction. The CVR was calculated in the ICH-free hemisphere for the ICH group and in bilateral hemispheres for healthy controls.

In addition to CVR measurement, conventional MRI markers of SVD were evaluated using the Standards for Reporting Vascular Changes on Neuroimaging criteria as previously described (13). WMH was rated using the Fazekas scale in periventricular and deep brain regions (14). Higher scores were considered as the overall WMH scale in each patient. WMH volume was also calculated as described previously (5). The location and number of CMBs were evaluated in SWI (15). Enlarged perivascular spaces (EPVS) were defined as sharply delineated structures in T2-weighted imaging measuring <3 mm following the course of perforating or medullary vessels (16). The number of EPVS (on the side of the brain with more severe involvement) was counted in the basal ganglia and centrum semiovale, and we pre-specified a dichotomized classification of the degree of EPVS as high (score > 20) or low (score ≤ 20) (16). Lacunes were defined as round or ovoid, subcortical, fluid-filled cavities (with similar signal to CSF) between 3 and 15 mm in diameter (13, 17). To estimate the generalized SVD burden, the total SVD score was determined using four neuroimaging markers according to established literature (18, 19). One point was awarded to the score for each of the following: the presence of lacunes, microbleeds, over 20 dilated perivascular spaces in the basal ganglia, and moderate to severe overall WMH (Fazekas score 2 or 3).

### Statistical Analysis

Categorical variables are shown as percentages, and continuous variables are shown as the mean ± standard deviation. Demographic, clinical, and neuroimaging features were compared between patients with hypertensive ICH and healthy controls using an independent two-sample *t*-test for continuous variables and Fisher's exact test for categorical factors as appropriate. In multivariable regression analyses, we investigated the independent associations between hypertensive ICH and regional CVR with further adjustment for age and sex.

Within the ICH group, the association between CBF changes and conventional MRI markers was assessed using correlation analyses, which were also adjusted for age and sex using partial correlation analysis. Because of skewed distribution of WMH volume in our participants, WMH volume was log transformed for the correlation analysis. Statistical analyses were performed using SPSS version 25 (SPSS Inc., Chicago, IL). All tests of significance were 2-tailed with a threshold for significance of *p* < 0.05.

## Results

Twenty-one hypertensive ICH patients (mean age 62.5 ± 11.3 years, 76.2% male) and 10 healthy controls (mean age 66.1 ± 6.0 years, 30% male) were recruited (Table 1). All patients completed dipyridamole challenge tests. Three patients experienced dizziness (*n* = 2) or chest discomfort (*n* = 1) after the study, and their symptoms resolved after the administration of aminophylline. Hypertensive ICH patients (13 strictly deep hemorrhage, 8 mixed lobar and deep hemorrhage) more often had a history of chronic hypertension (100% vs. 50%, *p* = 0.001) and showed poorer cognitive performance on the Montreal Cognitive Assessment (22.7 ± 5.4 vs. 27.8 ± 2.7, *p* = 0.022) than healthy controls. Hypertensive ICH was also significantly associated with conventional image markers of hypertensive SVD, including deep CMB (76.2% vs. 0%, *p* < 0.001) and higher volume of WMH (7.0 ± 5.6 vs. 2.6 ± 2.0 mL, *p* = 0.007).

**Table 1:** Demographics in patients with spontaneous intracerebral hemorrhage and controls.

	**ICH (*n* = 21)**	**Control (*n* = 10)**	***p*-value**
**Male, %**	16 (76.2%)	3 (30.0%)	0.021
Age, y	62.5 ± 11.3	66.1 ± 6.0	0.354
**Hypertension**	21 (100%)	5 (50.0%)	0.001
^*^Mean arterial pressure, mmHg	100 ± 16	96 ± 12	0.512
Mini-mental status examination	25.8 ± 4.1	27.8 ± 2.6	0.237
**Montreal cognitive assessment**	22.7 ± 5.4	27.8 ± 2.7	0.022
Diabetes, %	4 (19.0%)	2 (20.0%)	1.000
Dyslipidemia, %	9 (42.9%)	6 (60.0%)	0.458
eGFR, mL/min/1.73 m^2^	72.7 ± 17.1	85.2 ± 14.6	0.059
Presence of lobar CMB	13 (61.9%)	2 (20.0%)	0.054
**Presence of deep CMB**	16 (76.2%)	0 (0%)	<0.001
**WMH volume, mL**	7.0 ± 5.6	2.6 ± 2.9	0.007
Enlarged perivascular space (≥20)			
Centrum semiovale	5 (23.8%)	4 (40.0%)	0.417
Basal ganglia	6 (28.6%)	1 (10.0%)	0.379
Presence of lacune	10 (47.6%)	2 (20.0%)	0.240

*Values are mean (±standard deviation) or number (percentage)*.

* *Blood pressure was measured before CVR study*.

*CMB, cerebral microbleed; eGFR, estimated glomerular filtration rate; WMH, white matter hyperintensity*.

**Figure 1:**
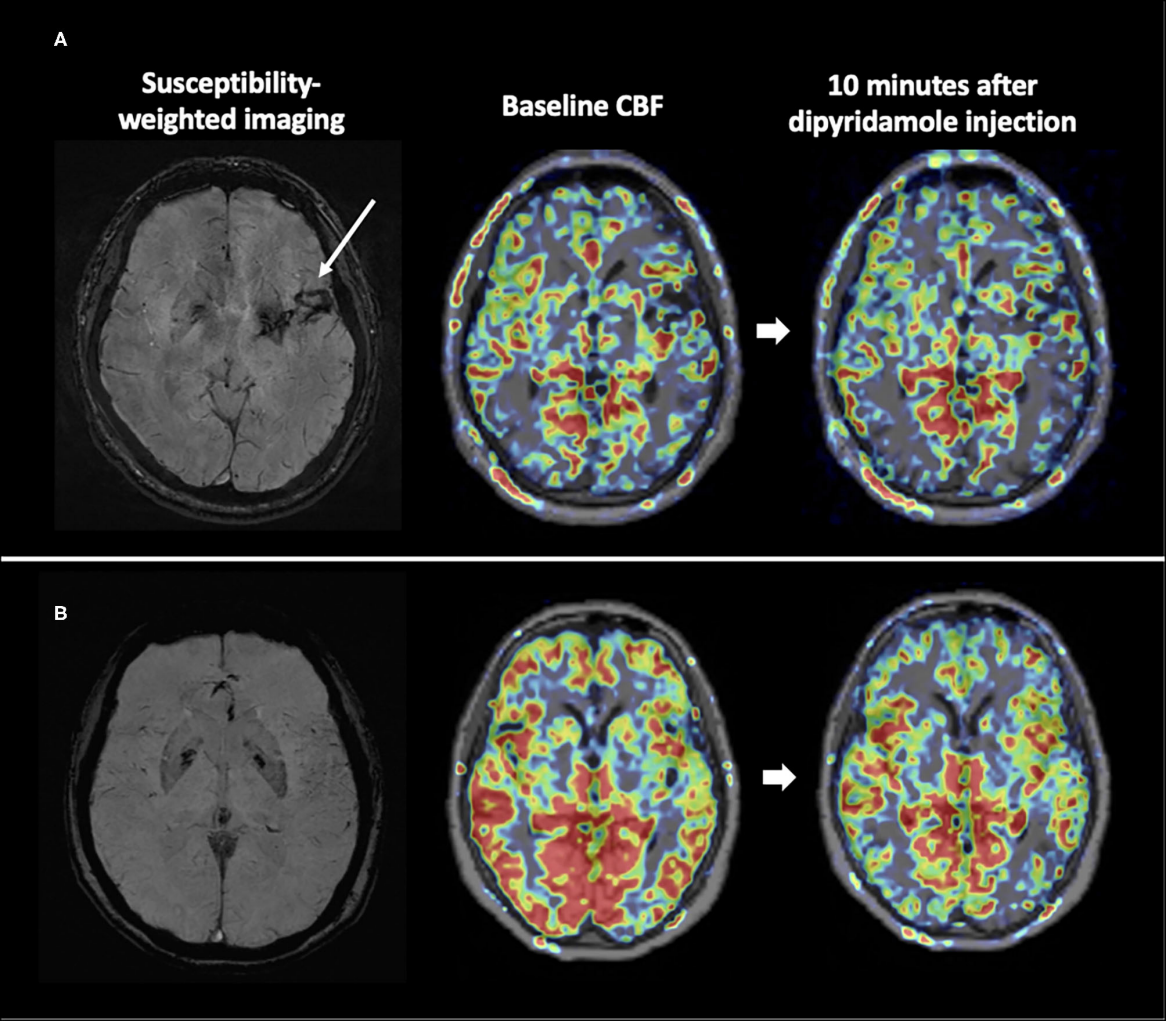
CVR maps from representative subjects. The ICH patient **(A)** had little reduction in CBF after vaso-constrictive challenge compared to the healthy counterpart **(B)**.

Figure 1 shows representative images from the ICH and healthy control groups obtained during the dipyridamole challenge test. There was no difference for the baseline regional or global relative CBF between ICH patients and normal controls (all p > 0.05). Compared to control subjects, hypertensive ICH patients showed reduced CBF changes in the basal ganglia (CBF reduction 22.4 ± 22.7% vs. 41.7 ± 18.3%, *p* = 0.026), the frontal lobe (15.1 ± 11.9% vs. 26.6 ± 9.9%, *p* = 0.013), and the temporal lobe (14.7 ± 11.1% vs. 26.2 ± 10.0%, *p* = 0.010) (Table 2). These differences remained significant in multivariable models after adjustment for age and sex. On the other hand, there was no significant difference in CBF reduction in the cerebral white matter, parietal or occipital lobes between the ICH group and healthy controls.

**Table 2:** Comparison of ASL perfusion reduction in spontaneous intracerebral hemorrhage and controls.

	**ICH (*n* = 21)**	**Control (*n* = 10)**	***p*-value**	^*^Adjusted *p*-value
**Basal ganglia, %**	22.4 ± 22.7	41.7 ± 18.3	0.026	0.020
**White matter, %**	23.9 ± 15.7	28.7 ± 13.9	0.419	0.385
**Frontal lobe, %**	15.1 ± 11.9	26.6 ± 9.9	0.013	0.025
**Temporal lobe, %**	14.7 ± 11.1	26.2 ± 10.0	0.010	0.031
Parietal lobe**, %**	19.2 ± 17.3	25.3 ± 11.2	0.318	0.340
Occipital lobe**, %**	21.2 ± 15.2	25.7 ± 12.9	0.422	0.114

* *Adjusted for age and sex*.

**Figure 2:**
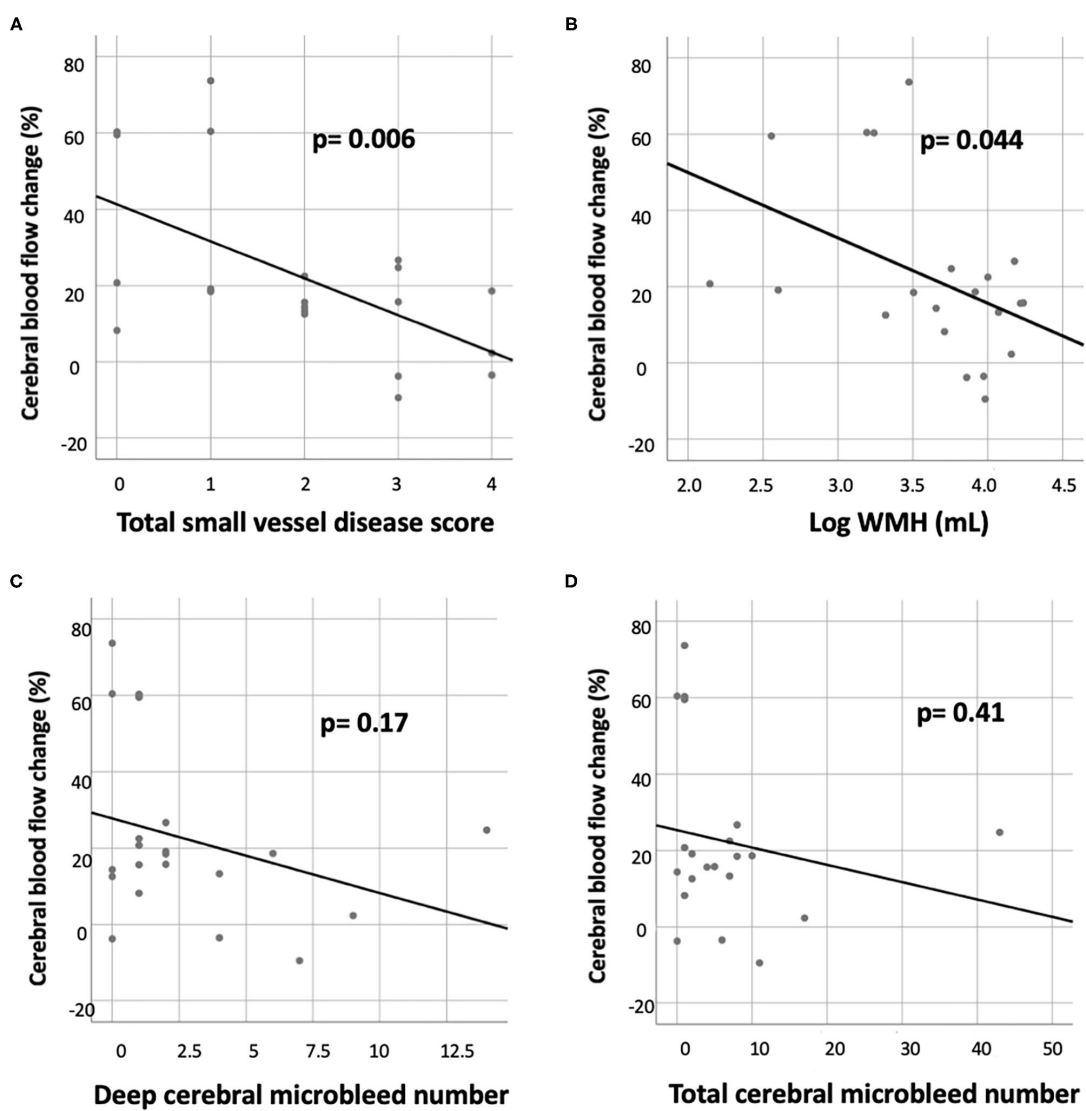
Correlation of CBF reduction in the basal ganglion with various small vessel disease markers in the ICH group, including **(A)** total small vessel disease score, **(B)** white matter hyperintensity (WMH) volume (log transformed), **(C)** deep cerebral microbleed number, and **(D)** total cerebral microbleed number.

Within the ICH group, the CBF reduction in the basal ganglia was correlated with the total SVD score (*R* = −0.58, *p* = 0.006) and log WMH volume (*R* = −0.44, *p* = 0.044), but not with deep CMB or the total number of CMBs (Figure 2). The correlation between CBF reduction in the basal ganglia and the total SVD score remained significant after adjustment for age and sex (*p* = 0.021). There was no significant correlation between CBF reduction in the frontal or temporal lobes and conventional MRI markers for SVD after adjustment for age and sex (all *p* > 0.05).

## Discussion

This study shows that hypertensive ICH patients are associated with decreased CVR in the basal ganglia, frontal lobe, and temporal lobe, compared to age-matched healthy controls. In hypertensive ICH patients, the CVR impairment in the basal ganglia was significantly associated with cerebral SVD burden according to conventional MRI, including WMH severity and total SVD score. Our study provides a novel but feasible methodology to measure CVR in SVD. Furthermore, the observed relationship between regional CVR impairment and hypertensive ICH further supports our hypothesis that not only deep but also cortical vessels are affected in advanced hypertensive SVD.

Previous studies have shown an association between conventional MRI markers of SVD and reduced CVR. For example, the severity of WMH was inversely correlated with CBF and CVR (20, 21). Likewise, patients with lacunar stroke were found to have elevated vascular stiffness and reduced CVR (22, 23). However, the total burden of SVD, which is represented by an ordinal scale of the total SVD score, had only limited sensitivity in predicting the neurological outcomes of patients (18, 24). Further investigation is warranted to determine whether CVR assessment can offer additional prognostic value to current SVD imaging markers.

Spontaneous ICH in the basal ganglion or thalamus is mostly attributed to hypertensive SVD (4), and there is evidence of arteriolosclerotic change involving small perforators in the deep cerebral regions (3). In this study, anterior circulation including the basal ganglia, frontal lobe, and temporal lobe showed impaired CVR in patients with hypertensive SVD. This finding may represent regions with small vessels that are vulnerable to chronic hypertension. However, it has been shown that CVR is generally lower in the posterior circulation than the anterior circulation in physiological conditions, which may be why our results showed no significant CVR difference between groups in occipital regions (25, 26).

Impaired CVR has also been reported in other forms of SVD, such as cerebral amyloid angiopathy and cerebral autosomal dominant arteriopathy with subcortical infarcts and leukoencephalopathy (CADASIL) (27, 28). In line with the literature, we showed that CVR by dipyridamole challenge is associated with WMH and the total SVD score. Thus, we hypothesized that both deep and cortical arterioles that are susceptible to hypertensive SVD do not dilate sufficiently regarding increased flow demand, leading to a hypoxic environment in the supplying territories and ultimately resulting in the formation of WMH (29).

One of the strengths of our study is the use of ASL MRI for CVR imaging. Transcranial Doppler ultrasound and position emission tomography (PET) have also been used in CVR imaging. However, MRI outperforms these other CVR modalities due to its superior spatial resolution compared to PET and less operator dependency compared to Doppler ultrasound. The major advantage of ASL is that it can be quantitative (30), whereas conventional perfusion MRI techniques can only show relative changes in the cerebral perfusion parameters (31). ASL enables us to assess both regional and global cerebral perfusion without using contrast material.

CVR imaging usually requires a vaso-reactive challenge to be implemented during the perfusion imaging process. The most used method is the hypercapnia challenge, which can be induced with CO_2_ inhalation or breath-holding, leading to vaso-dilatation and increased cerebral blood flow (32). Another commonly used method is injecting acetazolamide, a carbonic anhydrase inhibitor, which elicits cerebral vaso-dilatation by lowering pH value and increasing carbonic acid in the intracranial arteries (33, 34). In this study, we used intravenous dipyridamole to induce cerebral vaso-constriction as a vaso-reactive challenge during CVR imaging. Despite being a vasodilator for the coronary artery, dipyridamole decreases cerebral blood flow via adenosine-induced hyperventilation and resultant hypocapnia (35). The dipyridamole challenge test has been a widely implemented procedure in diagnosing coronary artery disease and has few risks, meaning that it could be a safe and feasible approach for CVR imaging.

Our study has several limitations. First, there was a significant sex difference between ICH patients and healthy controls, which was probably due to the recruitment process. However, the CVR in the basal ganglia, frontal lobe, and temporal lobe remained significant after adjustment for sex and age. Second, the sample size was limited (*n* = 31). The results in this proof-of-concept study warrants further trials investigating CVR in SVD with a larger cohort. Third, a few patients (*n* = 3) experienced dizziness or chest discomfort after dipyridamole administration, but their symptoms were tolerable and completely ameliorated after the administration of aminophylline. There are other alternatives for vaso-reactive challenges, such as CO_2_ inhalation or acetazolamide administration. However, CO_2_ inhalation requires sophisticated instruments to maintain the arterial CO_2_ level, and acetazolamide stimulation may also cause discomfort, such as numbness, paresthesia, and headache (33). Lastly, we did not monitor arterial CO_2_ level during the dipyridamole challenge. Therefore, the observed difference in CVR may be biased by individual responsiveness of hypocapnic hyperventilation to dipyridamole despite weight-based dosing.

In conclusion, we have demonstrated that patients with advanced hypertensive SVD showed impaired CVR in the basal ganglia, frontal lobe, and temporal lobe, and that reduced CVR is significantly associated with conventional MRI markers of SVD. Our CVR measurement approach may provide a surrogate marker to evaluate the efficacy of medication and/or interventional treatment from a functional perspective.

## Data Availability Statement

The original contributions presented in the study are included in the article/supplementary material, further inquiries can be directed to the corresponding author/s.

## Ethics Statement

The studies involving human participants were reviewed and approved by The Institutional Review Board, National Taiwan University Hospital. The patients/participants provided their written informed consent to participate in this study.

## Author Contributions

B-CL: project concept and design, data collection, imaging analysis, data analysis, and write up. H-HT: project concept and design, patient enrollment, data collection, imaging analysis, data analysis, and write up. A-HH and L-KT: patient enrollment and critical revisions. Y-LL: data collection and imaging analysis. Y-FC: project concept and design, data collection, and critical revisions. W-CW: project concept and design, data collection, imaging analysis, and critical revisions. All authors contributed to the article and approved the submitted version.

## Conflict of Interest

The authors declare that the research was conducted in the absence of any commercial or financial relationships that could be construed as a potential conflict of interest.
